# Development and Validation of a Kidney-Transplant Specific Measure of Treatment Burden

**DOI:** 10.1186/s12882-022-02923-3

**Published:** 2022-09-03

**Authors:** Elizabeth C. Lorenz, Tanya M. Petterson, Isabella Zaniletti, Kandace A. Lackore, Bradley K. Johnson, Martin L. Mai, Sumi S. Nair, Andrew J. Bentall, Kathleen J. Yost, David T. Eton

**Affiliations:** 1 William J Von Liebig Center for Transplantation and Clinical Regeneration, Rochester, MN USA; 2 Division of Nephrology and Hypertension, Mayo Clinic, Rochester, MN USA; 3 Division of Clinical Trials and Biostatistics, Department of Quantitative Health Sciences, Mayo Clinic, Rochester, MN USA; 4 Quantitative Health Sciences, Mayo Clinic, Scottsdale, AZ USA; 5 Department of Transplantation, Mayo Clinic, Jacksonville, FL USA; 6 Mayo Clinic Transplant Center, Phoenix, AZ USA; 7 Department of Quantitative Health Sciences, Mayo Clinic, Rochester, MN USA; 8 Division of Health Care Delivery Research, Robert D. and Patricia E. Kern Center for the Science of Health Care Delivery, Mayo Clinic, Rochester, MN USA

**Keywords:** Treatment Burden, Kidney Transplant, Quality of Life, Adherence, Chronic Kidney Disease

## Abstract

**Background:**

Treatment burden refers to the work involved in managing one’s health and its impact on well-being and has been associated with nonadherence in patients with chronic illnesses. No kidney transplant (KT)-specific measure of treatment burden exists. The aim of this study was to develop a KT-specific supplement to the Patient Experience with Treatment and Self-Management (PETS), a general measure of treatment burden.

**Methods:**

After drafting and pretesting KT-specific survey items, we conducted a cross-sectional survey study involving KT recipients from Mayo Clinic in Minnesota, Arizona, and Florida. Exploratory factor analysis (EFA) was used to identify domains for scaling the KT-specific supplement. Construct and known-groups validity were determined.

**Results:**

Survey respondents (*n* = 167) had a mean age of 61 years (range 22–86) and received a KT on average 4.0 years ago. Three KT-specific scales were identified (transplant function, self-management, adverse effects). Higher scores on the KT-specific scales were correlated with higher PETS treatment burden, worse physical and mental health, and lower self-efficacy (*p* < 0.0001). Patients taking more medications reported higher transplant self-management burden.

**Conclusions:**

We developed a KT-specific supplement to the PETS general measure of treatment burden. Scores may help providers identify recipients at risk for nonadherence.

**Supplementary Information:**

The online version contains supplementary material available at 10.1186/s12882-022-02923-3.

## Background

Kidney transplantation (KT) is the treatment of choice for patients with end-stage kidney disease (ESKD) because it is associated with better patient survival, cost, and quality of life (QOL) compared to dialysis [1, 2]. Unfortunately, there is a significant organ shortage, and over 90,000 patients in the United States are currently waiting for a KT [3, 4]. Once a patient receives a deceased donor kidney transplant, it only lasts an average of 12 years [5]. In a recent position statement, the National Kidney Foundation highlighted extending transplant longevity as a critical research priority [2]. A major contributor to KT failure is nonadherence [6]. Nonadherence to immunosuppressive therapy has been observed in approximately 25% of KT recipients who face a complex regimen of medications, laboratory monitoring, and medical appointments [6]. However, adherence involves more than just taking medications. The World Health Organization defines adherence as a multidimensional construct which also involves instituting dietary and lifestyle changes recommended by health care providers [7].

A potentially unrecognized contributor to nonadherence in KT recipients is treatment burden. Treatment burden refers to the work involved in taking care of one’s health and the impact of that work on personal well-being [8]. The work involved in taking care of one’s health is multifaceted and includes not only taking medications, but also seeking and understanding medical information, health monitoring, maintaining medical appointments, diet, and exercise [9, 10]. Treatment burden has been associated with nonadherence, difficulty navigating the healthcare system, and decreased QOL in non-transplant patients [8, 11–14]. Fortunately, treatment burden appears amenable to interventions like simplifying medication regimens and increasing home-based care [15].

**Fig. 1:**
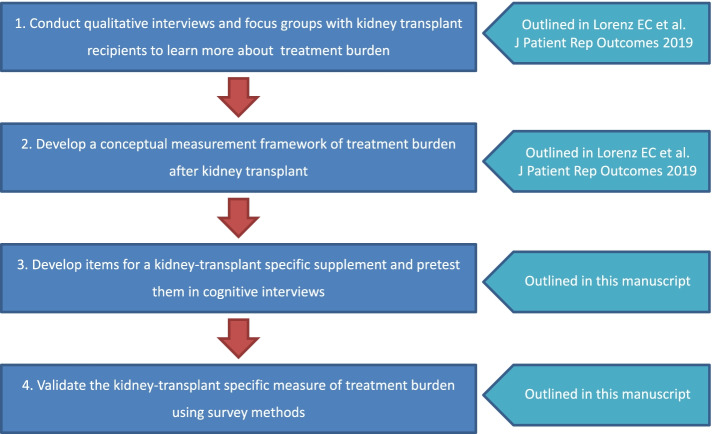
Process of developing a kidney-transplant specific measure of treatment burden

Despite the clinical significance and potential reversibility of treatment burden, it is understudied in patients with chronic kidney disease (CKD), including KT recipients. Currently, there are no validated measures of treatment burden in KT recipients. Our overall goal is to adapt the Patient Experience with Treatment and Self-Management (PETS) [8], a measure of treatment burden developed in patients with multiple chronic diseases, for use after KT. We previously completed the first two steps toward this goal (Fig. 1). Specifically, we developed a conceptual framework of treatment burden after KT by conducting qualitative interviews and focus groups with KT recipients [16]. We found that the domains of the general PETS measure were relevant and applicable after KT, but that KT recipients also experience KT-specific burden. The purpose of this study to develop and validate a new KT-specific measure of treatment burden by adding a module of supplementary items to the core PETS measure.

## Methods

### Study participants

Our study was approved by the Mayo Clinic Institutional Review Board (#19–001,693). Inclusion criteria included: 1) 18 years of age or older and 2) received a KT at Mayo Clinic in Minnesota, Mayo Clinic Arizona, or Mayo Clinic Florida. Exclusion criteria included: 1) neuropsychiatric condition causing patient to be unable to provide consent, 2) non-English speaking, and 3) failed KT defined as return to dialysis or relisting for KT. Oral consent and Health Insurance Portability and Accountability authorization were obtained.

### Cognitive pre-testing

Based on the KT-specific issues of treatment burden we identified in our prior qualitative study [16], we drafted 14 items for a supplement to the PETS. Cognitive pre-testing of these 14 items was conducted with KT recipients from Mayo Clinic in Minnesota, Mayo Clinic Arizona, or Mayo Clinic Florida between 2/2020 and 4/2020. We conducted two rounds of interviews either in person or via telephone. Six participants participated in the first round of interviews, and 5 participants participated in the second round of interviews. During each round, the interviewer asked participants to complete the module of items, comment on their understanding of each item, and rate the importance of each item to their post-KT health using a zero to ten scale. After the first round of cognitive interviews, we removed two items focused on tension between the participant’s living kidney donor and themselves, because the participants did not consistently feel that this issue was important and because it is covered within the “relationships with others” scale of the PETS (version 2.0). After the first round of cognitive interviews, we also added two items regarding immunosuppression side effects because participants repeatedly endorsed the importance of this issue. We also made minor modifications to the wording of the items based on patients’ responses during both rounds of cognitive interviews. Changes made to the items of the KT-specific supplement during cognitive pre-testing are outlined in Supplemental Table 1.

**Table 1:** Demographics of survey respondents

Characteristics of Respondents	*N* = 167
Age (years), mean (range)	60.9 (21.7–86.1)
Male, N (%)	93 (55.7)
Race, N (%)	
White	138 (84.7)
Black/African-American	9 (5.5)
Asian	6 (3.7)
American Indian/Alaskan Native	4 (2.5)
Mixed race (more than one race)	4 (2.5)
Other	2 (1.2)
Hispanic/Spanish/Latino ethnicity, N (%)	7 (4.2)
Marital status, N (%)	
Never married	15 (9.1)
Married	124 (75.2)
Living with partner	7 (4.2)
Separated, divorced, widowed	19 (11.5)
Education level, N (%)	
8^th^ grade or less	1 (0.6)
Some high school	1 (0.6)
High school graduate/GED	28 (16.8)
Some college/technical degree	57 (34.1)
College graduate	50 (29.9)
Advanced degree	30 (18.0)
Work status, N (%)	
Full-time employed	58 (35.2)
Part-time employed	15 (9.1)
Homemaker	6 (3.6)
Retired or unemployed	68 (41.2)
On disability or leave	18 (10.9)
Current yearly household income, N (%)	
Less than $20,000	16 (10.4)
$20,000 to $29,999	13 (8.4)
$30,000 to $39,999	6 (3.9)
$40,000 to $59,999	23 (14.9)
$60,000 to $79,999	23 (14.9)
$80,000 to $99,999	21 (13.6)
$100,000 or more	52 (33.8)
Current living situation, N (%)	
Living in a home	150 (90.4)
Living in an apartment	11 (6.6)
Assisted living/nursing home	1 (0.6)
Homeless	1 (0.6)
Other	3 (1.8)
Including yourself, current number people residing in your home, N (%)	
2	92 (60.9)
3	19 (12.6)
4 or more	16 (10.6)
Time from kidney transplant (years), mean (range)	4.0 (0.3–24.2)
Donor type, N (%)	
Deceased	55 (32.9)
Living related	42 (25.1)
Living unrelated	70 (41.9)
Prior kidney transplant, N (%)	16 (9.6)
History of pre-transplant dialysis, N (%)	93 (55.7)
Cause of end-stage renal disease, N (%)	
Glomerulonephritis	48 (28.7)
Diabetes	30 (18.0)
Polycystic kidney disease	36 (21.6)
Other or unknown	53 (31.7)
Estimated glomerular filtration rate (GFR) (ml/min/1.73 m2), mean (range)	49.0 (15–90)
Comorbidities, N (%)	
Diabetes	51 (30.5)
Hepatitis	2 (1.2)
Glaucoma	4 (2.4)
Depression	38 (22.8)
Asthma	10 (6.0)
Osteoarthritis	9 (5.4)
Hyperlipidemia	117 (70.1)
Hypertension	151 (90.4)
Coronary artery disease	28 (16.8)
Cancer	39 (23.4)
Congestive heart failure	9 (5.4)
Inflammatory arthritis	7 (4.2)
Number of comorbidities, N (%)	
1	43 (25.7)
2	49 (29.3)
3	48 (28.7)
4	27 (16.2%)
Median number of conditions	3.0
Number of medications, mean (range)	11.5 (3.0–21.0)
Immunosuppression, N (%)	
Tacrolimus	150 (89.8)
Cyclosporine	8 (4.8)
Belatacept	6 (3.6)
Sirolimus	3 (1.8)
Prednisone maintenance, N (%)	122 (73.1)
History of acute rejection, N (%)	40 (24.0)
Estimated glomerular filtration rate (ml/min/1.73 m^2^), mean (range)	49 (15–90)
Smoking history, N (%)	
Actively smoking	3 (1.8)
Prior smoker	63 (38.0)
Never smoker	100 (60.2)
Adherence to medications, N (%)	
Always take all medications	158 (94.6)
Usually take all medications (85% of time)	8 (4.8)
Sometimes take all medications (< 80% of time)	1 (0.6)
How often do you have problems learning about your medical condition because of difficulty understanding written information? N (%)	
All of the time	2 (1.2%)
Most of the time	0 (0.0%)
Some of the time	13 (7.8%)
Little of the time	32 (19.2%)
None of the time	120 (71.9%)
How confident are you filling out forms by yourself? N (%)	
Always	127 (76.0%)
Often	24 (14.4%)
Sometimes	8 (4.8%)
Occasionally	3 (1.8%)
Never	5 (3.0%)
How often do you have trouble understanding medical information spoken to you by doctors or nurses? N (%)	
All of the time	4 (2.4%)
Most of the time	3 (1.8%)
Some of the time	17 (10.2%)
Little of the time	49 (29.3%)
None of the time	94 (56.3%)

### Survey administration

The Mayo Clinic Survey Research Center prepared the survey packet for mailing. Each packet included a cover letter, a postage-paid return envelope and the following surveys: our 14-item KT-specific module of supplemental questions; the 60-item general PETS (version 2.0) [8] assessing treatment burden; the 10-item Patient-Reported Outcomes Measurement Information System (PROMIS) Global-10 (version 1.0) assessing physical and mental health [17]; the 25-item Burden, Symptoms/Problems and Effects of Kidney Disease scales of the Kidney Disease Quality of Life Short-Form (KDQOL-SF) (version 1.3) [18–20] assessing health-related quality of life; the 5-item side effects and 3-item convenience subscales of the Treatment Satisfaction Questionnaire for Medication (TSQM) [21] assessing difficulties taking medications and medication side effects; and the 8-item Perceived Medical Condition Self-Management Scale (PMCSM) [22, 23] assessing self-efficacy in managing one’s health. Education, work status, number of prescription medications, medication adherence [24], convenience of healthcare services and financial distress related to healthcare costs were assessed using single items. Subjective health literacy was assessed using three items, including difficulty understanding written information, spoken information, and filling out forms [25].

To have 80% power to detect an effect size of 0.5 at the one-tailed 5% significance level and calculate known-groups validity, we determined that a sample size of at least 102 participants would be needed. Based on prior experience surveying KT recipients [26] and validating the PETS [8], we expected a survey response rate of 35% and determined we would invite 300 patients to complete the survey packet. Survey packets were thus mailed to a random sample of 300 patients who had received KTs within the past 10 years, including 150 patients from Mayo Clinic in Minnesota, 75 patients from Mayo Clinic Arizona, and 75 patients from Mayo Clinic Florida. To maximize response rate, a second mailing was sent to nonrespondents one month after the first mailing. Patients who still did not respond received a phone call reminder from the Mayo Clinic Survey Research Center, and the survey packet was resent if needed. An additional 122 survey packets were distributed to patients presenting for routine transplant clinic appointments at Mayo Clinic in Minnesota. The period of survey recruitment was from 10/2020 through 6/2021. Participants who completed a survey packet received remuneration ($10).

### Data analysis

**Fig.2:**
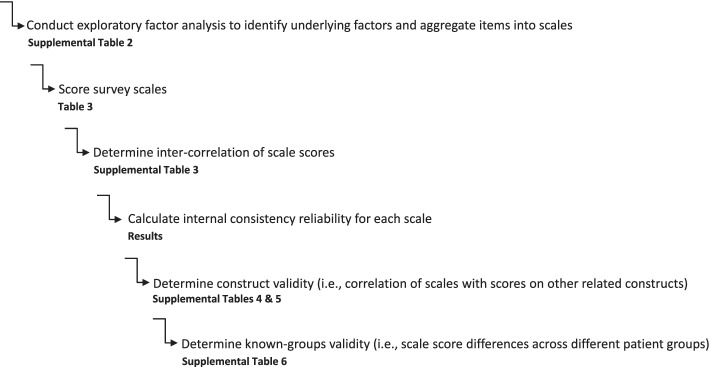
Steps involved in validating the kidney-transplant specific measure of treatment burden

Data were summarized by mean, median, standard deviation and range for continuous variables, and counts and percentages for categorical variables. Demographics, medications, and information about the patient’s medical history were abstracted from the electronic medical record. Estimated glomerular filtration rate within 12 months of survey completion was estimated using the Chronic Kidney Disease Epidemiology Collaboration equation [27]. All analyses except for exploratory factor analysis outlined below were conducted in SAS 9.4. An overview of subsequent steps involved in validating the KT-specific measure of treatment burden is outlined in Fig. 2.

### Exploratory factor analysis

An exploratory factor analysis (EFA) was performed on items within the KT-specific supplement to determine whether there were underlying patterns in the data that could justify aggregation of items into domain scales. Factors were extracted based on two standard criteria: eigenvalues > 1.0 and examination of the scree plot. Principal axis factoring (PAF) was used to extract factors followed by a non-orthogonal oblique rotation (promax) to facilitate interpretation of individual factors, and final item communalities (*h*^2^), the common variance of an item accounted for by the factors, were calculated. Factor analysis was conducted in IBM SPSS 28.0. Based on the EFA results, the KT-specific items were aggregated into scales.

### Scale scoring

KT-specific supplement scales were scored using previously published PETS scoring methods [8]. Briefly, raw scale scores were converted to a 0–100 scale with higher scores indicating higher burden. Scale scores were determined as long as > 50% of the items within the scale were non-missing. Items rated as not applicable were treated as missing. Correlations among scale scores were also examined using Spearman’s rank-order correlations.

### Reliability and validity

After determining scaling of the KT-specific supplement items using EFA, internal consistency reliability was determined by calculating Cronbach’s α coefficients for each scale with α’s ≥ 0.70 indicating adequate reliability. Construct validity was determined by correlating scores of the KT-specific supplement scales with the general treatment burden scores of the PETS and other related constructs (i.e., adherence, quality of life, etc.). Construct validity of the KT-specific scales was supported if at least a moderate correlation was identified with the PETS scales and scores on other related constructs using Spearman rank-order correlations (*ρ* ≥ 0.30) [28]. Known-groups validity was determined by comparing groups of patients predicted to have different levels of treatment burden (i.e., patients with diabetes compared to patients without diabetes) using independent sample t-tests.

## Results

### Patient characteristics

Overall, 167 participants completed a survey distributed via mail or in clinic. Of the 300 surveys distributed via mail, 125 participants responded (41.7%), including 66 of 150 participants from Mayo Clinic in Minnesota (44.0%), 27 of 75 from Mayo Clinic Arizona (36.0%), and 32 of 75 from Mayo Clinic Florida (42.7%). Of the 122 surveys distributed in clinic at Mayo Clinic in Minnesota, an additional 42 participants responded (34.4%). Demographics of all 167 participants are outlined in Table 1. Mean participant age was 60.9 (12.7) years, and the median time from KT was 4.0 (1.2–5.4) years. Overall, 55.7% of respondents were male, 84.7% were white, 82% finished some college or obtained a technical degree, 75.2% were married, and 41.2% were retired or unemployed.

### Item scores

Scores from the 14 individual items within the KT-specific supplement are displayed in Table 2. Two items contained large amounts of missing data (≥ 25% of participants responding “does not apply to me”) and were therefore excluded. This included Q9 (“I have felt like I would like to communicate more with my kidney donor or the family of the person who donated my kidney”) and Q10 (“My dialysis graft or fistula [area of arm where needles went during hemodialysis] has bothered me”).

**Table 2:** Scores from original items within the kidney-transplant specific supplement

Item^1, 2^	N	Mean (SD)	Score range	Not Applicable or Missing
Q1. I have been concerned my KT is losing function	160	2.98 (0.96)	1.00–4.00	7
Q2. I have been confident that I am taking good care of my KT	163	1.67 (0.72)	1.00–4.00	4
Q3. I have been concerned that I will have to started dialysis in the future	154	3.04 (0.96)	1.00–4.00	13
Q4. I have been concerned about my body rejecting my KT	160	2.88 (0.93)	1.00–4.00	7
Q5. I have been concerned that my original kidney disease will return and affect the function of my KT	146	2.90 (0.97)	1.00–4.00	21
Q6. I have been confident that I will never do anything that will hurt my KT	163	1.79 (0.79)	1.00–4.00	4
Q7. I feel responsible for my kidney transplant doing well	163	1.48 (0.57)	1.00–3.00	4
Q8. I feel that I know enough about the person who donated my kidney	156	1.79 (1.03)	1.00–4.00	11
Q9. I have felt like I would like to communicate more with my kidney donor or the family of the person who donated my kidney	126	2.66 (1.01)	1.00–4.00	41
Q10. My dialysis graft or fistula has been bothering me	76	2.92 (1.04)	1.00–4.00	91
Q11. I have been concerned about developing an infection of any type	161	2.61 (0.98)	1.00–4.00	6
Q12. I have been concerned about developing a cancer of any type	162	2.69 (0.89)	1.00–4.00	5
Q13. I have had difficulty taking my anti-rejection medications as directed	164	3.55 (0.72)	1.00–4.00	3
Q14. I have been bothered by side effects of my anti-rejection medications	163	2.87 (1.03)	1.00–4.00	4

^1^All items use the following response scale: 1-strongly agree; 2-agree; 3-disagree; 4-strongly disagree; 5-not applicable; ^2^ The PETS measure, including all adaptations, derivations, and translations are protected by copyright, © 2020 Mayo Foundation for Medical Education and Research. All Rights Reserved. Queries regarding any aspect of this work should be addressed to the corresponding author

### Exploratory factor analysis 

An EFA performed on the remaining 12 items revealed three factors with corresponding eigenvalues of 4.06, 1.85, and 1.07. The three factors accounted for 46% of the total variance. Two items were removed from the factor model due to very low item communalities (*h*^2^ < 0.25): “I feel I know enough about the person who donated my kidney” (Q8) and “I have had difficulty taking my anti-rejection medications as directed” (Q13).

The EFA was re-run on the remaining 10 items and once again revealed three factors, with corresponding eigenvalues of 3.94, 1.73, and 1.01. The three factors accounted for 53% of the total variance. An oblique promax rotation was used to facilitate interpretation of the loadings of the extracted factors. Factor loadings and item communalities appear in Supplemental Table 2. The first factor included items assessing concern about loss of KT function, return to dialysis, rejection, or recurrent disease (Q1, 3, 4, 5) and was labelled “transplant function.” The second factor included items assessing confidence in one’s ability to take care of the KT and feeling responsible for the KT (Q2, 6, 7) and was labelled “transplant self-management.” The third factor included concern about side effects of immunosuppression (Q11, 12, 14) and was labelled “transplant adverse effects.” Final item communalities ranged from 0.36 to 0.78.

Based on the EFA results, the KT-specific items were aggregated into three domain scales (i.e., transplant function, transplant self-management, and transplant adverse effects) with standard PETS scoring used to determine domain scale scores [8]. Spearman’s correlations of each of the three KT-specific scales are displayed in Supplemental Table 3. Mean scale scores of the PETS measure, the newly derived KT-specific scales, and the other measures included in the survey are displayed in Table 3.

**Table 3:** Responses to PETS, the kidney-transplant specific supplement scales, and other surveys

	N	Mean (SD^1^)	Score range^2^
PETS^3^ scales			
Medical information	162	17.4 (16.5)	0.0–71.4
Medications	159	13.0 (16.5)	0.0–92.9
Medical appointments	158	15.1 (15.8)	0.0–58.3
Monitoring health	157	22.1 (22.1)	0.0–100.0
Diet	166	32.6 (20.0)	0.0–100.0
Exercise or physical therapy	166	39.8 (24.3)	0.0–100.0
Relationships with others	164	12.1 (16.6)	0.0–87.5
Medical and health care expenses	162	32.3 (24.9)	0.0–100.0
Difficulty with health care services	156	26.8 (19.5)	0.0–90.5
Role/social activity limitations	162	16.2 (20.4)	0.0–100.0
Physical/mental fatigue	161	21.8 (21.3)	0.0–90.0
Bother due to reliance on medicine	159	13.4 (22.5)	0.0–100.0
Bother due to medicine side effects	159	23.9 (28.9)	0.0–100.0
KT-specific supplement scales			
Transplant function	158	35.1 (26.1)	0.0–100.0
Transplant self-management	164	21.7 (18.7)	0.0–77.8
Transplant adverse effects	162	42.6 (24.8)	0.0–100.0
PROMIS Global-10^4^			
Global Physical Health	156	49.9 (8.8)	26.7–67.7
Global Mental Health	164	50.0 (9.0)	21.2–67.6
KDQOL-SF^5^			
Burden of kidney disease	165	77.5 (23.6)	0.0–100.0
Symptoms/problems of kidney disease	166	85.6 (13.6)	25.0–100.0
Effects of kidney disease	166	85.9 (16.5)	6.3–100.0
TSQM^6^			
Side effects	158	80.1 (26.8)	0.0–100.0
Convenience	81	81.2 (18.0)	22.2–100.0
PMCSM^7^			
Self-Management	166	33.7 (6.0)	14.0–40.0

^1^Standard deviation; ^2^Scores for surveys range from 0 (lowest) to 100 (highest) with PMCSM ranging from 8 (lowest) to 40 (highest); ^3^Patient Experience with Treatment and Self-Management; ^4^Patient-Reported Outcomes Measurement Information System (T-Score); ^5^Kidney Disease Quality of Life Short-Form; ^6^Treatment Satisfaction Questionnaire for Medication; ^7^Perceived Medical Condition Self-Management Scale

### Reliability, construct validity, and known-groups validity

Internal reliabilities for the three KT-specific supplement scales were the following: Cronbach’s α = 0.83 for transplant function, α = 0.72 for transplant self-management, and α = 0.66 for transplant adverse effects. In terms of construct validity, all three scales of the KT-specific supplement were positively and significantly correlated with the treatment burden scores of the PETS scales (Supplemental Table 4). Higher scores on the transplant function scale were most strongly correlated with higher burden on the medical and healthcare expenses scale of the PETS (*ρ* = 0.36), higher scores on the transplant self-management scale were most strongly correlated with higher burden on the monitoring health scale of the PETS (*ρ* = 0.47), and higher scores on the transplant adverse effects scale were most strongly correlated with higher burden on the medication side effects bother scale of the PETS (*ρ* = 0.52).

Similarly, higher scores on the KT-specific supplement scales were associated with scores on established measures of general physical and mental wellbeing, kidney disease-specific quality of life, medication satisfaction, and self-efficacy (Supplemental Table 5). Higher scores on the KT-specific supplement scales were significantly correlated with worse physical and mental health (PROMIS-10); worse burden, symptoms/problems, and effects of kidney disease (KDQOL-SF); more bother due to medication side effects (TSQM side effects); and lower self-efficacy for managing chronic illness (PMCSM). Most of the correlations (86%) were of medium size or greater (*ρ* ≥ 0.30) supporting construct validity of the KT-specific supplement scales.

Scores on the KT-specific supplement scales were also compared across groups of patients to assess known-groups validity (Supplemental Table 6). We found that patients with diabetes, patients with more comorbidities, patients taking more medications, and patients transplanted > 1 year ago had significantly higher burden scores on the transplant self-management scale. Patients with an eGFR < 30 ml/min/1.73 m^2^ had significantly higher burden scores on the transplant function scale and a trend toward significantly higher burden scores on the transplant adverse effects scale (54.2 ± 29.7 versus 32.1 ± 24.6, *p* = 0.004 and 54.9 ± 30.5 versus 41.6 ± 23.6, *p* = 0.07, respectively).

## Discussion

The purpose of this study was to develop and validate a KT-specific supplementary module to the PETS general measure of treatment burden. Based on our previously developed conceptual framework of treatment burden after KT [16], we drafted items for a KT-specific supplement and then pretested them during cognitive interviews. The supplementary items were pilot tested in a cross-sectional survey study of 167 KT recipients from three geographically dispersed Mayo Clinic transplant centers. Exploratory factor analysis supported three scales within the KT-specific supplement, including transplant function, transplant self-management, and transplant adverse effects. Internal reliability was acceptable (alpha ≥ 0.70) for the transplant function and transplant self-management scales, but slightly lower in the transplant adverse effects scale (alpha = 0.66), possibly due to qualitative differences in the adverse effects queried (i.e., development of another health condition versus medication side effects). Construct validity of our supplement was demonstrated by correlating scores on the three scales with scores of general treatment burden (i.e., the PETS) and other established measures of established constructs. Scores on several of the KT-specific scales were quite high relative to scores on the PETS suggesting that the supplement may provide additional information regarding treatment burden in KT recipients compared to the general measure.

Scores on the KT-specific supplementary scales were associated with differences in patient characteristics. We found that patients with diabetes, more comorbidities, and more medications reported significantly higher burden on the transplant self-management scale. Prior studies have also demonstrated an association between treatment burden, comorbidities, and medications [29–31]. KT recipients forced to manage multiple comorbidities and complex medication regimens may perceive a greater sense of responsibility and self-management burden. We also found that patients who received a KT > 1 year ago reported significantly higher burden on the transplant self-management scale. This higher self-management burden may be related to less frequent follow-up and guidance from transplant centers after the first post-transplant year. Our finding that patients with an eGFR < 30 ml/min/1.73 m^2^reported significantly higher burden on the transplant function scale is not surprising. Patients with reduced function may be aware that they are closer to needing to start dialysis or undergo retransplantation; KT recipients often view allograft failure as an outcome worse than death [32].

Although patients seen by nephrologists have been shown to be more medically complex, have more comorbidities, and take more medications than patients seen by other subspecialists [33], little is known about treatment burden in patients with CKD. The few studies conducted in this vulnerable population have shown that more than one-third of patients with pre-dialysis and dialysis-dependent CKD experience moderate to high treatment burden, and that treatment burden is associated with decreased quality of life and reduced social support [34, 35]. Even less is known about treatment burden after KT. KT recipients may be especially vulnerable to excessive treatment burden given their potentially fragmented healthcare and the long-term side effects and cost of immunosuppression.

Developing a KT-specific measure of treatment burden is an important initial step to improving treatment burden. Treatment burden has been shown to be associated with nonadherence in non-transplant patients [8, 36]. Thus, screening for treatment burden after KT could identify patients at high risk for nonadherence to immunosuppression. KT recipients experiencing high treatment burden might benefit from interventions shown to lessen treatment burden in patients with other chronic conditions, including simplified treatment regimens and increased home-based care [15]. Furthermore, a KT-specific measure of treatment burden could also serve as a benchmark in future clinical trials designed to improve adherence, QOL, and KT survival.

The study does have several limitations. First, response rates were below 50% at each of the three centers. This may have introduced a response bias. Treatment burden may have been underestimated if the most burdened KT recipients chose not to participate in the study. Second, although our respondents received KTs from three geographically dispersed transplant centers, all three were Mayo Clinic centers. Thus, results may be less generalizable to patients receiving transplants at other centers, including centers outside the United States. Interestingly, treatment burden as measured by the PETS has been found to be relevant to patients with chronic health conditions in other countries with different healthcare systems, including the United Kingdom and Norway [37–39]. Third, our cohort had relatively low representation of racial/ethnic minorities and the less formally educated, and future studies should include a more diverse cohort. Lastly, our study was conducted during the coronavirus disease of 2019 (COVID-19) pandemic which may have impacted our results. The pandemic may have increased treatment burden by limiting access to medical care potentially leading to exacerbations of medical conditions. Immunosuppressed KT recipients may have experienced higher burden related to worry about infection and adverse impacts of isolation on social, physical, and psychological function. Conversely, the pandemic may have lessened treatment burden by providing greater access to telemedicine-based care [40].

## Conclusions

We utilized our previous conceptual framework of treatment burden after KT [16] to develop and validate a KT-specific supplement to the PETS, a general measure of treatment burden in patients with multiple chronic conditions [8]. The supplement contains three KT-specific scales: transplant function, transplant self-management, and transplant adverse effects. Our analyses provided support for the reliability, construct, and known-groups validity of these scales. Patients with reduced renal allograft function may be at especially high risk of burden. A KT-specific measure of treatment burden may promote identification of recipients at risk for nonadherence and graft loss, allow for modification of treatment regimens and ultimately improve patient-centered care, QOL, adherence, and long-term KT survival.

## Supplementary Information

**Additional file 1: Supplemental Table 1.** Changes made to items of the kidney-transplant specific supplement during cognitive pre-testing^1^. **Supplemental Table 2.** Rotated factor loadings and final item communalities for the kidney-transplant specific supplement. **Supplemental Table 3.** Spearman’s rank order correlations for kidney-transplant specific supplement scales generated by exploratory factor analysis. **Supplemental Table 4.** Spearman’s rank-order correlations for kidney-transplant specific supplement scales with PETS^1^ scales. **Supplemental Table 5.** Spearman’s rank-order correlations for kidney-transplant specific supplement scales with other survey measures. **Supplemental Table 6.** Comparison of scores for kidney-transplant specific supplement scales among different clinical groups^1^.

## Data Availability

The de-identified datasets generated and analyzed during this study are not publicly available due to privacy concerns but are available from the corresponding author on reasonable request. The PETS measure, including all adaptations, derivations, and translations are protected by copyright, © 2020 Mayo Foundation for Medical Education and Research. All Rights Reserved. Queries regarding any aspect of this work should be addressed to the corresponding author.

## Abbreviations

KT: Kidney Transplantation
ESKD: End-Stage Kidney Disease
QOL: Quality of Life
CKD: Chronic Kidney Disease
PETS: Patient Experience with Treatment and Self-Management
PROMIS: Patient-Reported Outcomes Measurement Information System
KDQOL-SF: Kidney Disease Quality of Life instrument Short-Form
TSQM: Treatment Satisfaction Questionnaire for Medication
PMCSM: Perceived Medical Condition Self-Management Scale
EFA: Exploratory Factor Analysis
