# Supplementary material for: Development and Validation of a Kidney-Transplant Specific Measure of Treatment Burden
Source: BMC Nephrol. 2022 Sep 3;23:301. doi: 10.1186/s12882-022-02923-3 (PMC9440455; doi:10.1186/s12882-022-02923-3)
Supplement: Supplementary file 1 — Additional file 1: Supplemental Table 1. Changes made to items of the kidney-transplant specific supplement during cognitive pre-testing1. Supplemental Table 2. Rotated factor loadings and final item communalities for the kidney-transplant specific supplement. Supplemental Table 3. Spearman’s rank order correlations for kidney-transplant specific supplement scales generated by exploratory factor analysis. Supplemental Table 4. Spearman’s rank-order correlations for kidney-transplant specific supplement scales with PETS1 scales. Supplemental Table 5. Spearman’s rank-order correlations for kidney-transplant specific supplement scales with other survey measures. Supplemental Table 6. Comparison of scores for kidney-transplant specific supplement scales among different clinical groups1. [file 12882_2022_2923_MOESM1_ESM.docx]

Supplemental Table 1. Changes made to items of the kidney-transplant specific supplement during cognitive pre-testing^1^

| Original questions | Modifications made after first round of cognitive interviews | Modifications made after second round of cognitive interviews |
| --- | --- | --- |
| I have been bothered by my living donor trying to tell me how to take care of myself. | (item removed) |  |
| There is tension between me and my living kidney donor. | (item removed) |  |
| I have been worried about my kidney transplant losing function. |  | I have been *concerned* about my kidney transplant losing function. |
| I have been confident that I am taking good care of my kidney transplant. |  |  |
| I have been fearful that I will have to start dialysis in the future. |  | I have been *concerned* that I will have to start dialysis in the future. |
| I have been worried about my body rejecting my kidney transplant. |  | I have been *concerned* about my body rejecting my kidney transplant. |
| I have been worried that my original kidney disease will return and affect the function of my kidney transplant. |  | I have been *concerned* that my original kidney disease will return and affect the function of my kidney transplant. |
| I have been confident that I will never do anything that will hurt my kidney transplant. |  |  |
| I have felt guilty about receiving another person’s kidney. | *I feel responsible for my kidney transplant doing well.* |  |
| I feel that I know enough about the person who donated my kidney. |  |  |
| I have felt like I would like to communicate more with my kidney donor or the family of the person who donated my kidney. |  |  |
| My dialysis graft or fistula has bothered me. | My dialysis graft or fistula *(area of the arm where needles went during hemodialysis)* has bothered me. |  |
| I have been worried about developing an infection. | I have been worried about developing an infection *of any type*. |  |
| I have been worried about developing cancer. | I have been worried about developing a cancer *of any type*. |  |
|  |  | *I have had difficulty taking my anti-rejection medications as directed.* |
|  |  | *I have been bothered by side effects of my anti-rejection medications.* |

^1^The PETS measure, including all adaptations, derivations, and translations are protected by copyright, © 2020 Mayo Foundation for Medical Education and Research. All Rights Reserved. Queries regarding any aspect of this work should be addressed to the corresponding author.

Supplemental Table 2. Rotated factor loadings and final item communalities for the kidney-transplant specific supplement

| Item | Factor 1  (Transplant Function) | Factor 2  (Transplant Self-Management) | Factor 3  (Transplant Adverse Effects) | Communality (*h*^2^) |
| --- | --- | --- | --- | --- |
| Q4 | .79 | .06 | .02 | 0.624 |
| Q3 | .75 | -.04 | .08 | 0.668 |
| Q1 | .66 | -.19 | -.02 | 0.525 |
| Q5 | .53 | -.04 | .24 | 0.507 |
| Q6 | .20 | .84 | -.24 | 0.775 |
| Q2 | -.20 | .62 | .12 | 0.427 |
| Q7 | -.13 | .61 | .18 | 0.368 |
| Q11 | .12 | .15 | .74 | 0.616 |
| Q12 | .17 | .11 | .59 | 0.465 |
| Q14 | -.04 | -.20 | .54 | 0.361 |

Supplemental Table 3. Spearman’s rank-order correlations for kidney-transplant specific supplement scales generated by exploratory factor analysis

|  | Transplant  Function | Transplant Self-Management | Transplant  Adverse Effects |
| --- | --- | --- | --- |
| Transplant Function | -- | 0.37  p < 0.0001 | 0.51  p < 0.0001 |
| Transplant Self-Management | 0.37  p < 0.0001 | -- | 0.18  p = 0.02 |

Supplemental Table 4. Spearman’s rank-order correlations for kidney-transplant specific supplement scales with PETS^1^ scales

|  | Transplant  Function | Transplant  Self-Management | Transplant  Adverse Effects |
| --- | --- | --- | --- |
| Medical Information | 0.15  p = 0.06 | 0.40  p < 0.0001 | 0.22  p = 0.005 |
| Medications | 0.29  p = 0.0003 | 0.42  p < 0.0001 | 0.19  p = 0.02 |
| Medication Reliance Bother | 0.29  p = 0.0003 | 0.37  p < 0.0001 | 0.38  p < 0.0001 |
| Medication Side Effects Bother | 0.31  p = 0.0001 | 0.30  p = 0.0001 | 0.52  p < 0.0001 |
| Medical Appointments | 0.25  p = 0.002 | 0.42  p < 0.0001 | 0.24  p = 0.002 |
| Monitoring Health | 0.25  p = 0.002 | 0.47  p < 0.0001 | 0.27  p = 0.0007 |
| Diet | 0.27  p = 0.0005 | 0.41  p < 0.0001 | 0.27  p = 0.0004 |
| Exercise or Physical Therapy | 0.30  p = 0.0001 | 0.33  p < 0.0001 | 0.29  p = 0.0002 |
| Relationships With Others | 0.33  p < 0.0001 | 0.32  p < 0.0001 | 0.37  p < 0.0001 |
| Medical and Healthcare Expenses | 0.36  p < 0.0001 | 0.32  p < 0.0001 | 0.31  p < 0.0001 |
| Difficulty With Healthcare Services | 0.29  p = 0.0004 | 0.41  p < 0.0001 | 0.31  p < 0.0001 |
| Role and Social Activity Limitations | 0.21  p = 0.01 | 0.26  p = 0.0008 | 0.30  p = 0.0002 |
| Physical and Mental Fatigue | 0.26  p = 0.001 | 0.34  p < 0.0001 | 0.38  p < 0.0001 |

^1^Patient Experience with Treatment and Self-Management

Supplemental Table 5. Spearman’s rank-order correlations for kidney-transplant specific supplement scales with other survey measures

|  | Transplant  Function | Transplant Self-Management | Transplant  Adverse Effects |
| --- | --- | --- | --- |
| PROMIS^1^ Global-10 Physical Health | -0.22  p = 0.007 | -0.42  p < 0.0001 | -0.38  p < 0.0001 |
| PROMIS^1^ Global-10 Mental Health | -0.40  p < 0.0001 | -0.58  p < 0.0001 | -0.35  p < 0.0001 |
| KDQOL^2^ Burden of Kidney Disease | -0.40  p < 0.0001 | -0.36  p < 0.0001 | -0.46  p < 0.0001 |
| KDQOL^2^ Symptoms/Problems of Kidney Disease | -0.22  p = 0.005 | -0.34  p < 0.0001 | -0.46  p < 0.0001 |
| KDQOL^2^ Effects of Kidney Disease | -0.33  p < 0.0001 | -0.33  p < 0.0001 | -0.46  p < 0.0001 |
| TSQM^3^ Side Effects | -0.32  p < 0.0001 | -0.29  p = 0.0002 | -0.56  p < 0.0001 |
| TSQM^3^ Convenience | -0.14  p = 0.21 | -0.34  p = 0.002 | -0.19  p = 0.10 |
| PMCSM^4^ | -0.36  p < 0.0001 | -0.50  p < 0.0001 | -0.36  p < 0.0001 |

^1^Patient-Reported Outcomes Measurement Information System; ^2^Kidney Disease Quality of Life; ^3^Treatment Satisfaction Questionnaire for Medication; ^4^Perceived Medical Condition Self-Management Scale

Supplemental Table 6. Comparison of scores for kidney-transplant specific supplement scales among different clinical groups^1^

|  | Diabetes | | | Upper quartile of comorbidities (≥ 4) | | | Upper quartile of medications (≥ 15) | | | Transplanted < 1 year ago | | | eGFR^2^ < 30 ml/min^/^1.73 m^2^ | | |
| --- | --- | --- | --- | --- | --- | --- | --- | --- | --- | --- | --- | --- | --- | --- | --- |
|  | No  (n=116) | Yes  (n=51) | p-value | No  (n=119) | Yes  (n=48) | p-value | No  (n=121) | Yes  (n=44) | p-value | No  (n=136) | Yes  (n=31) | p-value | No  (n=137) | Yes  (n=18) | p-value |
| Transplant  Function | 35.2 ± 28.0 | 34.8 ± 20.8 | 0.71 | 37.4 ± 27.0 | 29.4 ± 23.2 | 0.09 | 35.2  ± 26.0 | 35.3 ± 27.5 | 0.90 | 35.4 ± 25.9 | 34.0 ± 27.3 | 0.70 | 32.1 ± 24.6 | 54.2 ± 29.7 | 0.004 |
| Transplant Self-Management | 19.3 ± 18.1 | 27.3 ± 18.8 | 0.006 | 18.8 ± 17.6 | 29.0 ± 19.5 | 0.001 | 19.5 ± 18.1 | 27.3 ± 19.4 | 0.02 | 22.8 ± 18.8 | 16.8 ± 17.4 | 0.09 | 20.5 ± 18.4 | 28.5 ± 20.3 | 0.11 |
| Transplant Adverse Effects | 43.8 ± 24.8 | 39.9 ± 24.7 | 0.38 | 44.2 ± 24.9 | 38.9 ± 24.2 | 0.30 | 42.7 ± 25.2 | 43.2 ± 23.4 | 0.96 | 43.5 ± 25.1 | 39.1 ± 23.3 | 0.38 | 41.6 ± 23.6 | 54.9 ± 30.5 | 0.08 |

^1^Mean ± standard deviation; ^2^estimated glomerular filtration rate (n=155)
